# To Eat and Not Be Eaten: Modelling Resources and Safety in Multi-Species Animal Groups

**DOI:** 10.1371/journal.pone.0042071

**Published:** 2012-07-27

**Authors:** Umesh Srinivasan, Suhel Quader

**Affiliations:** 1 National Centre for Biological Sciences, Tata Institute of Fundamental Research, Bangalore, Karnataka, India; 2 Nature Conservation Foundation, Mysore, Karnataka, India; Vrije Universiteit, Netherlands

## Abstract

Using mixed-species bird flocks as an example, we model the payoffs for two types of species from participating in multi-species animal groups. Salliers feed on mobile prey, are good sentinels and do not affect prey capture rates of gleaners; gleaners feed on prey on substrates and can enhance the prey capture rate of salliers by flushing prey, but are poor sentinels. These functional types are known from various animal taxa that form multi-species associations. We model costs and benefits of joining groups for a wide range of group compositions under varying abundances of two types of prey–prey on substrates and mobile prey. Our model predicts that gleaners and salliers show a conflict of interest in multi-species groups, because gleaners benefit from increasing numbers of salliers in the group, whereas salliers benefit from increasing gleaner numbers. The model also predicts that the limits to size and variability in composition of multi-species groups are driven by the relative abundance of different types of prey, independent of predation pressure. Our model emphasises resources as a primary driver of temporal and spatial group dynamics, rather than reproductive activity or predation *per se*, which have hitherto been thought to explain patterns of multi-species group formation and cohesion. The qualitative predictions of the model are supported by empirical patterns from both terrestrial and marine multi-species groups, suggesting that similar mechanisms might underlie group dynamics in a range of taxa. The model also makes novel predictions about group dynamics that can be tested using variation across space and time.

## Introduction

Animal groups are ubiquitous in nature, and can be either relatively simple single-species groups, or more complex multi-species associations. Multi-species groups differ from single-species groups in certain key aspects. For instance, because resource requirements are likely to be more similar for conspecifics than heterospecifics, competition within multi-species groups is expected to be much lower compared with competition in single-species groups [1]. Further, multi-species group participants might introduce benefits or costs arising from specialised behaviours to other group members in terms of predator avoidance, foraging efficiency, or both [2]. Therefore, understanding multi-species groups requires an evaluation of costs and benefits to group participants arising from multiple simultaneously operating interactions such as competition, mutualism or commensalism.

Multi-species animal groups have been studied in a wide range of taxa, from spiders [3] and reef fish [4], to birds [5] and mammals, including cetaceans [6], primates [7] and ungulates ([8], review in [9]). The main benefits of multi-species grouping are widely held to be increased foraging efficiency and reduced predation risk [10]. However, despite a large body of literature on multi-species groups spanning almost 150 years [11], the theoretical framework of cost and benefit in multi-species groups has not been explored using formal mathematical models (as noted by [12], but see [13] for a graphical model of the mutualism between mongooses and hornbills).

As opposed to verbally stated hypotheses, which have hitherto guided multi-species group research, mathematical models enable making hidden assumptions explicit, and can be explored for testable predictions that might not be apparent in a verbal argument [14]. Such models, therefore, are useful tools in guiding research that attempts to tease out mechanisms underlying empirically observed patterns. We present a mathematical model of the benefits and costs of multi-species grouping, using mixed-species bird flocks (and the differences in behaviour and ecology of participant bird species) as an example. Our model incorporates two broad types of species – gleaners (poor at predator detection and capable of increasing resource access to salliers) and salliers (good at detecting predators, but incapable of providing foraging benefits to others through increased access to resources). The species roles we model have parallels in other multi-species animals groups, including fish [15], hornbills and mongooses [13], and primates [16].

In our model, the payoff to flock participants is the number of prey consumed when in flocks (of varying size and composition) relative to the number of prey consumed when solitary. Based on these payoffs, we examine variation in the ‘allowed’ size and composition of multi-species groups under conditions of differing availability of two resource types, and offer potential explanations for the diversity of multi-species group dynamics in time and space. Our model includes parameters that are intrinsic to species and potentially influence foraging facilitation and/or predator avoidance, as well as those related to the external habitat (i.e. prey abundance), that might be expected to influence multi-species grouping.

## Methods

### Model Formulation

Unlike for multi-species groups, the theoretical framework underlying associations in single-species group has been well developed [17], [18]. Single-species groups are similar to multi-species groups in some aspects (including individual roles – producers increase access to resources, and scroungers exploit increased resource access) and gleaners and salliers are indeed analogous to producers and scroungers in many ways. However, these two types of groups differ in several key aspects, and producer-scrounger models fall short of being applicable to multi-species groups. First, gleaners and salliers in multi-species groups feed on different types of prey, whereas producers and scroungers in single-species groups are assumed to exploit the same resource. Further, where the decision to be a producer or scrounger might be flexible, the foraging behaviour of gleaners and salliers is likely to be stereotypical. Finally, game theoretic approaches equalise pay-offs for producers and scroungers, which might not be a suitable approach when modelling different species likely to differ in their forage requirements.

### Species Characteristics

We characterize bird species participating in mixed-species flocks as gleaners and salliers (after [19]) based on their foraging behaviour. Gleaners are species that forage by searching for and picking insects and arthropods off substrates such as foliage. They are therefore likely to be poor at detecting predators [20], and good at disturbing (or flushing) prey from the substrates on which they forage [21]. Salliers, on the other hand, are species that continually scan their environment for prey from open perches and capture insects in flight. These species, because of their scanning behaviour, are expected to be good at predator detection (during scanning bouts but not when actually pursuing and capturing prey), and incapable of flushing prey [21], [22]. Salliers, however, can capture prey flushed by gleaners.

### Habitat Characteristics

Gleaners and salliers feed on different types of prey. We therefore included in our model two types of prey that could vary in abundance (and therefore in their rate of being encountered by birds). These were prey in vegetation and prey in flight. In our model, gleaners feed only on prey in vegetation. Salliers feed on prey in flight, but in the company of gleaners, can also access a certain proportion of prey from the vegetation that is flushed by gleaners. In contrast, gleaners can never access prey that is in flight, and therefore also lose access to any prey that they flush.

### Modelling Costs and Benefits

We modelled the costs and benefits to individuals participating in mixed-species flocks (henceforth, flocks) as the number of prey consumed when in a flock relative to the number of prey consumed when foraging alone. The number of prey consumed was modelled as a product of the time spent foraging and the prey capture rate. A net foraging benefit of flocking accrues to an individual when the number of prey consumed within a flock exceeds the number of prey consumed when foraging alone. The opposite situation results in a net foraging cost to joining a flock. Costs and benefits from flocking were examined separately for individual gleaners and salliers participating in flocks of various compositions. To generate these flocks, we varied the number of gleaners and salliers in the flock under different resource availability conditions, such that composition ranged from flocks of 1 gleaner and 1 sallier to those with *n_g_* gleaners and *n_s_* salliers.

### Modelling Time Spent Foraging through Vigilance Time

Our model assumes that the only demands on the time of an individual are foraging and avoiding predation, and that these are mutually exclusive. Therefore, in our model, the proportion of time spent foraging and the proportion of time spent avoiding predators sum to one. We further assume that the overall vigilance of a group of any size or composition equals solitary vigilance. In other words, any group should be as likely to detect a predator as a solitary individual. Therefore, if all individuals in a group contribute to vigilance, per capita investment in anti-predator behaviour in a group is lower than when solitary, allowing for more time to forage when in a group. We make a further simplifying assumption, that there is no ‘cheating’ in time spent vigilant by some individuals and consequently compensation for cheating by others. We further do not explicitly account for the relationship between the ‘dilution effect’ in flocks and the possibility that larger groups are more conspicuous to predators.

Given these assumptions, if *t_0_* is the proportion of time spent vigilant by a gleaner when foraging alone, the proportion of time a single gleaner spends avoiding predation from joining a group of *n* other individuals (*n_g_* additional gleaners and *n_s_* salliers, where n  =  *n_g_* + *n_s_*) is:


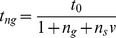


where *v*, or the ‘vigilance parameter’ is a measure of how much better a sallier is at detecting predators compared with a gleaner (or, the number of gleaners that are equivalent to one sallier in terms of predator detection) and is always greater than one. This parameter is best thought of as the probability of a sallier detecting and warning against a predator first relative to the probability of a gleaner detecting and warning against a predator first. In other words, if a sallier detects one out of every two predators, and a gleaner one out of every four, the sallier would be ‘twice as good’ a sentinel as a gleaner, and *v* would have a value of two. This, however, might not necessarily correlate with simple measures such as the ratio between the time spent scanning for predators by a sallier relative to the time spent scanning by a gleaner because of differences in scanning behaviour between the two species types. For instance, gleaner scanning might be less efficient than sallier scanning because the former tend to forage in denser vegetation compared with salliers, which are expected to have a wider field of vision.

The proportion time spent foraging is:


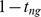


This formulation results in a geometric-like decline in vigilance with increasing group size, an effect empirically observed in several species [23]. For the sake of simplicity, and as a starting point to examine model predictions, we assume that the proportion of time spent vigilant by a solitary sallier is identical to the proportion of time spent vigilant by a solitary gleaner. Salliers can detect predators when scanning for prey; however, we do not account explicitly for this in our model. Rather, we retain the more tractable formulation under which foraging and vigilance are mutually exclusive for salliers as well as gleaners. This decision does not affect our conclusions materially: decreasing the proportion of time spent vigilant for solitary salliers does not affect the qualitative predictions of the model.

In parallel with the model for gleaners, above, the proportion of time spent in predator avoidance for an individual sallier in a flock of *n_g_* gleaners and *n_s_* additional salliers is.


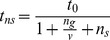


and the proportion time spent foraging is 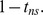


Our formulation of how vigilance changes with group size assumes not only that vigilance declines in a non-linear fashion with group size, but also that individuals in a group partition vigilance among themselves perfectly. The relationship between group size and vigilance appears to depend on on several factors [24], [25]. However, a review of 172 studies on vigilance and group size in birds found strong support for a negative relationship between group size and vigilance [23]. Some empirical studies have shown that individuals scan for predators independently of each other [26], [27], but we retain the ‘perfect partitioning’ formulation of vigilance for simplicity. Indeed, qualitative predictions of our model are not affected by devaluing the efficiency of each individual by any proportion (tested between 0.1 to 1), which is the expected manner in which vigilance might be affected if individuals in a group scanned for predators independently of one another.

### Modelling Prey Capture Rate through Search Time

Prey capture rate depends on search time (*s*) and handling time (*h*) as follows. Prey capture rate equals:


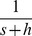


Assuming that handling time is different for salliers and gleaners, but constant for each, search time is the only parameter that can be affected by the number and nature of flock participants. In our model, search time for a solitary individual is the inverse of prey encounter rate, *k*, assuming that prey items are randomly encountered in the habitat and that prey encounter rate is proportional to the actual abundance of prey in the habitat. Search time therefore attains large values at low prey abundances (when prey encounter rate is also expected to be low) and approaches zero under conditions of superabundant prey. In our model, there are two prey types, prey in vegetation (accessible to gleaners) with an encounter rate *k_g_*, and prey in flight (accessible to salliers) with an encounter rate *k_s_*.

We also model the effect of prey flushing (the disturbance of prey from substrates by gleaners) on search time. Gleaners are modelled to have a probability of flushing an encountered prey item, p_g_ in some negligibly small time interval. Salliers do not flush prey. In a flock of *n_g_* gleaners and *n_s_* salliers, at any instant, the probability that at least one gleaner flushes prey equals:


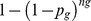


Therefore the expected number of prey flushed in any unit of time in a habitat where *k_g_* prey items are encountered per unit space equals 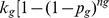. These flushed prey are accessible only to salliers as we assume that once flushed, prey escape predation by gleaners. Therefore, at any instant, the prey available to gleaners in a flock of *n_g_* gleaners and *n_s_* salliers is:


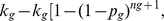


and the prey accessible to salliers in the same flock is: 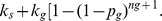


Therefore, search time for a gleaner joining a flock of *n_g_* additional gleaners and *n_s_* salliers in a habitat with randomly distributed prey encountered at *k_g_* and *k_s_* is:


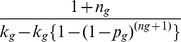


where the individual joining also flushes prey [therefore, the probability of at least a single prey item being flushed 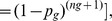 and total prey available is partitioned equally between all individuals with access to the prey. Search time for a sallier joining the same flock is:


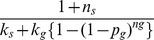


### Marginal Benefit

The marginal benefit for an individual joining a flock is the difference between the amount of prey consumed when foraging in a flock and the amount of prey consumed when foraging alone. Therefore, marginal benefit in a flock of *n* individuals is:


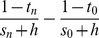


There is a net foraging gain from joining a flock when the marginal benefit is positive and a net cost when the marginal benefit is negative. Individuals facing a net cost would thus be expected to leave the flock and forage alone, whereas individuals gaining a net benefit should continue foraging within the flock.

### Model Parameters

The parameters in our model relate to intrinsic species characteristics as well as external habitat factors. In total, there are seven parameters in the model (Table 1). Information on the possible values of certain parameters is available from field studies and experiments, for instance the ‘vigilance factor’ from data in [21]. Despite this available information, to examine the robustness of our model, we explored our model by assigning a broader range of values to the parameters, given the following constraints: (1) the ‘vigilance factor’ (*v*) is always greater than one, to reflect that a sallier is better at detecting predators than a gleaner [28], and (2) the handling time (involving the pursuit, manipulation and consumption of prey already detected) for a sallier (*h_s_*) is always greater than the handling time for a gleaner (*h_g_*). This is because a sallier has to pursue prey, capture it in the air, return to its perch, process the prey, and then consume it. In contrast, a gleaner does not pursue its prey (which is typically immobile on foliage, bark or the ground), but only has to process and consume it once detected. We try to reflect the fact that gleaners do not usually pursue prey actively, but salliers always do, by constraining the handling time of salliers to be always greater than that of gleaners.

**Table 1:** Details of model parameters.

Parameter	Symbol	Gleaner	Sallier	Characteristics
Solitary vigilance time	*t_0_*	*–*	*–*	Proportion of time spent vigilant when foraging alone. Ranges from 0 to 1, no units. Tested with values from 0.1 to 0.5.
‘Vigilance factor’	*v*	*–*	*–*	Magnitude by which a sallier is a better than a gleaner in detecting predators. Always greater than 1, no units. Tested with values ranging from 2 to 15.
Handling time	*h*	*h_g_*	*h_s_*	Time taken to fully consume a prey item once it has been detected. Units of time/prey. Tested with values from 1 to 2 for *h_g_* and 2 to 4 for *h_s_*.
Prey flushing	*p*	*p_g_*	*–*	Probability of an encountered prey item being flushed in some finite time interval. Theoretically ranges from 0 (no encountered prey flushed) to 1 (all encountered prey flushed), no units. Tested with values ranging from 0.01 to 0.4.
Prey abundance	*k*	*k_g_*(prey invegetation)	*k_s_*(prey in flight)	Number of prey items encountered per unit time in the foraging range of a mixed-species flock. Theoretically ranges from 0 to infinity. Units in number of prey/time. Tested from 0 to 50.

### ‘Stable’ Group Sizes and Compositions

We define the size of a group as the total number of individuals within it, and its composition as its sallier to gleaner ratio. For any combination of prey abundances (in flight and in vegetation), more than one group (in terms of size and composition) can ‘emerge’ from the model in which gleaners and salliers receive simultaneous benefits. Where the number of individuals available to participate in flocks is limitless, the ‘stable’ group size and composition is likely to be the largest emergent flock in which every gleaner and sallier receives a net benefit from flocking. This is the largest possible flock in which both gleaners and salliers simultaneously receive benefits compared with solitary foraging. Beyond this flock size, either gleaners or salliers fare poorly compared with foraging alone, and would be expected to leave the flock. Also, solitary foragers would be expected to join smaller flocks to gain net benefits, thereby increasing flock size to this point. Note that the optimal flock size for any participant is likely to be smaller than the stable flock size [29].

## Results

### Model Predictions

**Figure 1:**
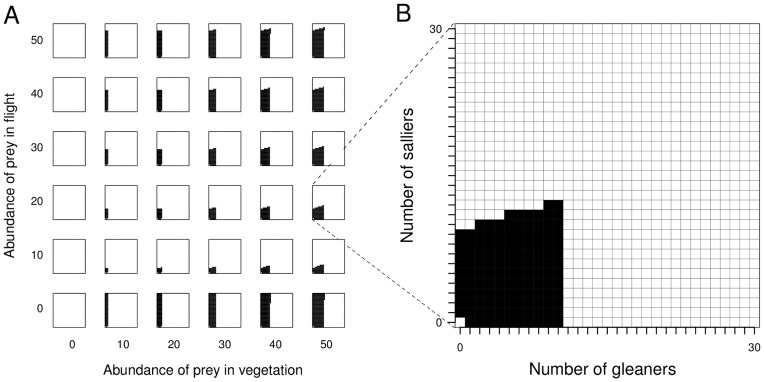
Resource abundance and emergent flocks. Change in mixed-species flock compositions with change in abundance of prey in vegetation and prey in flight (Fig. 1A). A close-up of one of the panels in Fig. 1A is shown in Fig. 1B. For each panel in Fig. 1A, the x-axis is the number of gleaners and the y-axis the number of salliers (both 0 to 30; see Fig. 1B). Each cell in Fig. 1B represents a flock with the corresponding number of gleaners and salliers. Cells coloured black represent flocks predicted from the model, in which each gleaner and sallier in the flock receives a net benefit over solitary foraging. White cells represent ‘forbidden’ combinations of gleaners and salliers (in which there is a net loss compared with solitary foraging). As an example, in Fig. 1B, the largest predicted flock consists of 10 gleaners and 12 salliers. All flocks with more than 10 gleaners are ‘forbidden’. As seen in Fig.1A, the range of predicted flocks increases with the abundance of prey. Values of other parameters: *p_g_* = 0.01, *v* = 2, *t_0_* = 0.2, *h_g_* = 1, *h_s_* = 2.

We varied the abundance of two types of prey (prey in vegetation, *k_g_*, and prey in flight, *k_s_*) in our model to examine the effect this has on the occurrence and composition of mixed-species flocks (Fig. 1). When there is no prey in the habitat (i.e. *k_g_* and *k_s_* = 0), as expected, flocks are not predicted by our model. Flocks are also not predicted when there are no prey in the vegetation in the habitat (i.e. *k_g_* = 0). When there are no prey in flight, and all prey are in vegetation, the model predicts flocks with high sallier to gleaner ratios (Fig. 1).

**Figure 2:**
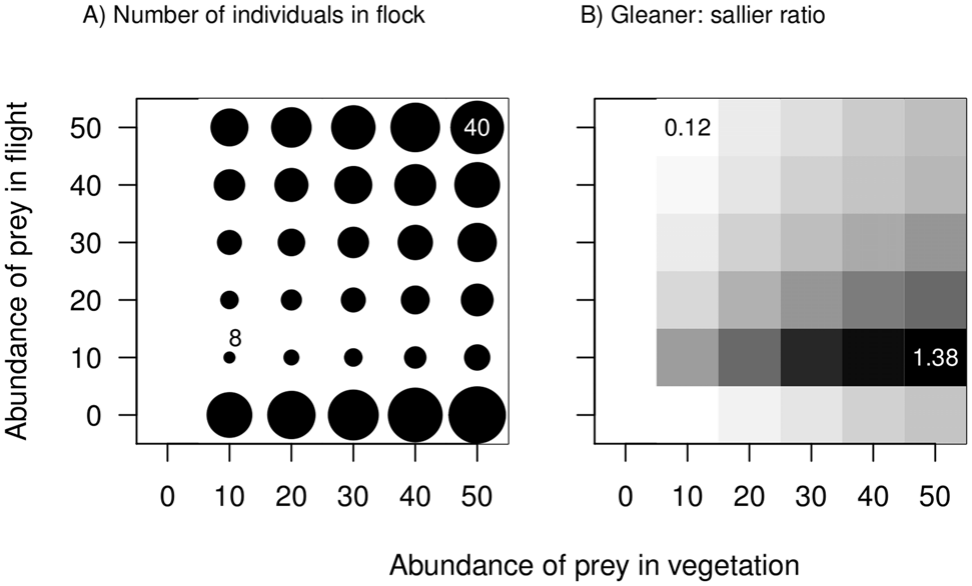
Resource abundance and flock size and composition. The relationship between ‘stable’ flock size (Fig. 2A), and composition in terms of gleaner:sallier ratio (Fig. 2B) in response to changes in prey abundance. Larger flocks are depicted by larger circles in Fig. 2A; The sizes of two flocks are printed to illustrate the relationship between circle size and flock size. Flocks with high gleaner to sallier ratios are darker in Fig. 2B; the gleaner to sallier ratios of two flocks are printed on the figure as a guide to the relationship between shade and flock composition. Parameter values are the same as in Fig. 1.

Despite a limitless pool of potential flock participants, our model predicts an upper limit on flock size, and this limit varies with prey abundance. Stable flock size increases with increasing abundance of prey in vegetation (Fig. 2A). For any given abundance of prey in vegetation, an increase in abundance of prey in flight also leads to an increase in the number of permitted compositions of mixed-species flocks (Fig. 1), as well as an increase in the stable flock size (Fig. 2A). Note that salliers dominate flocks when there is no prey in flight (Fig. 1), and ‘drop out’ when this prey becomes available. This accounts for the initial drastic fall in stable group size on addition of prey in flight (Fig. 2A; difference in stable group sizes between when prey in flight  = 0 and when prey in flight  = 5). In general, increasing the abundance of prey in vegetation leads to a decrease in the sallier to gleaner ratio in stable flocks (Fig. 2B), and for any given abundance of prey in vegetation, increasing the abundance of prey in flight increases the sallier to gleaner ratio in stable sized flocks (Fig. 2B). [Note that at low prey abundances, small flocks are predicted.] In such small flocks, the addition of even a single gleaner or sallier would cause large changes in sallier to gleaner ratios.

From our model, for any flock size and composition, a gleaner benefits from associating with salliers, and a sallier benefits from associating with gleaners. This is irrespective of both the species-specific as well as prey abundance parameter values. The addition of gleaners increases the benefit to salliers and the addition of salliers increases the benefit to gleaners. The incremental benefit, however, declines to reach an asymptote.

### Model Robustness

**Figure 3:**
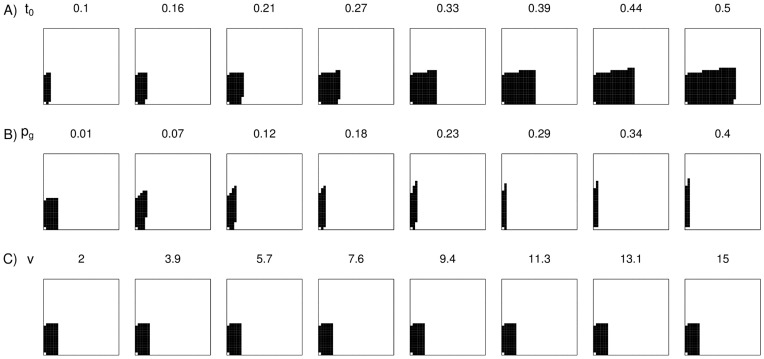
Testing model robustness. The relationship between parameter values and the proportion of predicted flock compositions on changing the values of (A) proportion of time spent by a solitary individual in avoiding predators (*t_0_*), (B) instantaneous probability of an encountered prey item being flushed, and (*p_g_*) (C) the ‘vigilance factor’ (*v*), which is a measure of how much better a sallier is than a gleaner in detecting predators. Example values of the parameters are shown. In each case, apart from the parameter value being tested, values of the other parameters were set as: *k_g_* = 25, *k_s_* = 25, *p_g_* = 0.01, *v* = 5, *t_0_* = 0.2, *h_g_* = 1, *h_s_* = 2.

The prediction that gleaners always benefit from associating with salliers, and salliers from associating with gleaners remains consistent for all explored values of all parameters in our model (summary of parameters in Table 1). The prey abundance parameters are the only parameters that influence whether mixed-species flocking is predicted to occur or not – i.e., the conditions under which mixed-species flocks are expected to form depend entirely on the values taken by the prey abundance parameters. Given particular values of the prey abundance parameters, changing the values of solitary vigilance time (*t_0g_*) and the instantaneous probability of a gleaner flushing prey (*p_g_*) alters flock composition. Increasing *t_0g_* results in an increase in permitted flock compositions (Fig. 3A), which arises from an increase in the number of gleaners that are ‘permitted’ to co-exist within the flock. An increase in *p_g_* increases the sallier to gleaner ratio in flocks and also reduces the number of permitted flock compositions (Fig. 3B). An increase in the value of the vigilance parameter (*v*) does not alter permitted flock compositions or gleaner: sallier ratios (Fig. 3C).

## Discussion

### Prey Abundance and Group Dynamics

The effect of prey abundance on flocking behaviour in our model offers insights into the potential role played by temporal and spatial variation in prey abundance in determining the occurrence, composition and stability of multi-species groups. When the abundance of prey in vegetation is zero or low and abundance of prey in flight is high, sallier-dominated flocks are predicted by our model (Fig. 1). Such conditions might occur during forest fires. During fires, the abundance of prey in vegetation might be low due to either destruction by fire, or because insects that are usually immobile take flight to escape being burnt. Fire therefore acts as a highly efficient ‘beater’ that flushes prey. Under such conditions, our model predicts aggregations of salliers. In accordance with this, sallier species such as drongos, which participate in mixed-species flocks, are known to forage together in large numbers at the edge of forest fires [30].

Mixed-species flocks with high sallier to gleaner ratios are predicted under conditions of complete absence of prey in flight (Fig. 2). Unlike gleaners, which can consume only prey in vegetation, salliers can consume, in addition to prey in flight, prey from vegetation flushed by gleaners. Under such conditions, associations of any number of salliers with one or more gleaners will be beneficial to salliers, because a sallier’s prey capture rate will always be greater than the rate when alone (i.e. zero). Based on our model, because gleaners lose prey to flushing even in the absence of salliers, associations with even an indefinitely high number of salliers would be beneficial to gleaners because salliers increase the time spent foraging by gleaners by decreasing their vigilance time. The only factor limiting group size when prey in flight are absent is competition between gleaners, which diminishes with increasing abundance of prey in vegetation. Thus, at very high abundances of prey in vegetation, and absence of prey in flight, a large number of possible flock compositions are permitted by our model (Fig. 1). Under such circumstances, groups would be expected to be much more variable, and possibly less cohesive than in other situations – group size and composition might be constrained simply by the availability of participants in the species pool.

### Temporal and Spatial Variation in Prey Abundance and Multi-species Group Dynamics

Seasonal patterns in flocking have hitherto been attributed to within-year variation in predation pressure [22] and breeding activity [31]. Our model suggests that, for multi-species groups, this view provides an incomplete picture at best. Instead, our results support the proposition that seasonal variation in multi-species groups (in terms of occurrence, composition or stability) is driven by the degree of seasonality in prey abundance [32], [33]. Seasonality in insect activity and abundance is known to correlate with several climatic variables, including temperature, day length, rainfall and humidity [34]. Variation in any of these climatic factors is likely to affect insect abundances through either direct effects or indirect trophic mechanisms like vegetation growth and phenology [35]. Further, because insects use cues such as temperature and rainfall to eclose [35], it is conceivable that prey in the habitat might shift from predominantly prey in vegetation to predominantly flying prey with a change in season. In Madagascar, for instance, the breeding season also corresponds with a peak in abundance of flying insects [36], [37], and changes in flock dynamics with season might simply be the result of differential prey availability, rather than because of breeding activities or change in predation risk.

Temperate and high-elevation areas are much more seasonal than the tropics, with wide annual variation in temperature, day length and rainfall. In the tropics, Asia, and especially monsoonal south and southeast Asia, shows high annual variation in rainfall, humidity and temperature (rainfall and temperature data from [38]). These are also areas where flocking is distinctly seasonal [39]–[43], with a peak in flocking during the cold winter months and the occurrence of less cohesive, shorter-lived or more compositionally variable flocks during the warm, wet season [31], [39], [43]. Such patterns are consistent with the predictions of our model. During the cold/dry season, when prey abundance is expected to be low, the model predicts a smaller range of ‘permitted;’ flocks and therefore low compositional variability (Fig. 2). In the wet/warm season, when prey abundance is likely to be higher, the model predicts a larger number of possible flock sizes and combinations and therefore potentially (within the constraints of abundance of participant species) flocks with high compositional variability (Fig. 1). In the neotropics, on the other hand, mixed-species insectivorous bird flocks occur all-year round and are compositionally similar irrespective of the time of year and breeding activity [44]–[48]. Permanent mixed-species flocks in the neotropics (occurring even during the breeding period) might be related to the relative aseasonality of the region [38], where the relative abundance of prey might not change significantly over the course of the year. A rigorous examination of the predictions relating to seasonality in the occurrence, size and cohesion of mixed-species groups would require comparative studies from sites with varying seasonal regimes, accompanied by data on seasonal variation in climatic factors and resource availability.

Spatial variation in prey abundance might also result in variation in multi-species group cohesiveness and activity. Differences in multi-species group dynamics between islands and between habitats, although widely thought to be because of differences in predation pressure [22], might instead arise from variation in prey activity and abundance. For instance, with increasing openness of habitats, although predator detectability does improve [22], habitat-driven changes in prey abundance (e.g., [49]) must not be dismissed in potentially influencing costs and benefits to flock participants. Variation in multi-species group composition and stability across different spatial scales in response to forage abundance has also been reported in primates [50] and reef fish [51].

Experiments and observational studies provide evidence that food availability influences flock dynamics (apparently independently of predation pressure) by increasing flocking propensity when resources occur at low abundances [52], [53]. Provisioning and prey depletion experiments that control for predation pressure, especially in more tractable multi-species systems such as reef fish, and possibly even frugivorous primates would be valuable in testing the mechanisms through which resource availability influences costs and benefits for different types of species in mixed-species groups. Conditions under which predation risk changes without change in resource availability or vice versa (for instance, predators on passage migration or outbreaks of insect prey) would also be suitable ‘natural experiments’ that might help understand mechanisms of multi-species group dynamics. Although predation risk (through changing proportion of time spent vigilant) can affect flock size and composition in our model (by mainly affecting the permitted number of gleaners in flocks; Fig. 3A), we show here that variation in resource abundance can have similar effects without changes in predation risk (Figs. 1 & 2). Further, changes in resource availability might have the potential to change both the permitted number of gleaners and salliers in mixed-species flocks.

### Model Predictions, and the Nature of Associations

A fairly obvious prediction from our model is that gleaners consistently prefer to associate with salliers, and that salliers prefer to associate with gleaners. The strong association between salliers and gleaners has been noted and quantified previously in mixed-species bird flocks [54], [55] as well as in other multi-species groups. In mixed schools of reef fish, substrate-disturbing species are associated with other species that gain feeding benefits from flushed prey [4]. In Amazonian tamarin groups as well, a follower species gains from prey disturbed by the leader species [16]. Thus, across several taxa, there seem to be associations between gleaner-analogues and sallier-analogues, the gleaners flushing prey, and salliers benefiting from increased access to flushed prey.

The association between gleaners and salliers has been hypothesised to result from the exchange of complementary benefits, each species playing a role that the other is unsuited for and providing benefits ordinarily unavailable to the other species [55]. Because gleaners search for prey on substrates such as sand, leaves and bark, they not only flush prey, but are also likely to be poor at detecting predators. This is because a gleaner engaged in searching for prey by visually examining substrates is expected to be incapable of simultaneously visually detecting predators. Therefore, gleaners must budget time specifically for vigilance. Salliers, which forage by capturing mobile prey, are capable of taking advantage of this flushed prey, and because of their habitat-scanning behaviour provide benefits of early warning against aerial predators to gleaners [21]. This verbal model suggests that in a gleaner-sallier association, gleaners should mainly benefit from an increase in time spent foraging (resulting from a reduction in time spent vigilant), and salliers should benefit mainly from an increase in prey capture rate. (Note that we do not explicitly model behaviours such as kleptoparasitism shown by some salliers, because the vast majority of sallier species in mixed-species flocks appear to benefit from flushed prey, rather than prey stolen from gleaners [21]).

In our model, gleaners benefit only from an increase in the proportion of time spent foraging, but not from higher prey capture rates, when associating with salliers. Also, salliers increase prey capture rates from associating with gleaners. Preliminary support for these expectations comes from both terrestrial and marine systems. Across 14 sites and 22 species, mixed-species bird flock leaders, which are invariably gleaners, did not show differences in prey capture rates within and outside of flocks. On the other hand, follower species, which are often salliers, significantly increased their prey capture rates in flocks [54]. In reef fish, a sallier-analogue follower species increased its prey capture rate when associating with a gleaner-analogue, whereas the prey capture rate for the gleaner species did not appear to change within and outside multi-species shoals [15]. However, such patterns might also depend on sampling methodology and may arise from an overall increase in time spent foraging and therefore more prey consumed over longer time periods.

The prediction that gleaners prefer to associate with salliers, and salliers with gleaners should be expected to lead to a conflict of interest between salliers and between gleaners in the group. Salliers should try and maximise the number of gleaners in the group and expel salliers, whereas gleaners should try to do the opposite: attract more salliers, and expel gleaners. We have not found documented evidence that gleaners try to expel gleaners and attract salliers, but several sallier species do appear to show behaviours (e.g. vocal mimicry) that attract gleaners. In Sri Lanka, the Greater Racket-tailed Drongo (*Dicrurus paradiseus*), a passerine bird, mimics the calls of other species, especially that of the Orange-billed Babbler (*Turdoides rufescens*), a gregarious gleaner. This mimicry attracts other species and initiates mixed-species flock, and drongos mimic more when alone than when within a mixed-species flock [26]. The same species of drongo in the Nicobar Islands might also use mimicry, but here to attract mammals [56]. Drongos also mimic the calls of predators of flocking species (such as sparrowhawks), which may be a manipulative way of elevating the threat perceived by other species in the flock. This might make gleaners artificially inflate the importance of foraging in the company of drongos to gain sentinel benefits that they actually do not require. Prior studies of mimicry in drongos (e.g., [57]) attribute the mimicking of predators by other birds to be alarm signals in response to predators. We propose an alternative explanation, that mimicry of predators might be a mechanism for manipulating the behaviour of other species for the benefit of the drongo. Manipulative behaviours such as vocal mimicry by sallier species, e.g. drongos, should occur at highest rates when prey in vegetation are abundant but flying prey are scarce. Under these conditions, drongos would be expected to feed primarily on prey flushed by gleaners. Mimicry levels should decline as the abundance of flying prey increases, because gleaners would be expected to have a declining effect on prey availability. Diurnal and seasonal variation in the abundance of flying prey could be used to test this prediction.

To our knowledge, our model is the first mathematical exploration of the dynamics of multi-species grouping, and points to unifying mechanisms influencing multi-species group dynamics across a range of terrestrial and marine taxa. Specifically, we develop and explore the role of resource availability and its fluctuation as a driver of multi-species group dynamics, which has received much less attention than the predation hypothesis. We also outline testable predictions from our model that would be valuable in examining variation (or its absence) in multi-species group dynamics across space and time.
